# Lessons from a decade of individual-based models for infectious disease transmission: a systematic review (2006-2015)

**DOI:** 10.1186/s12879-017-2699-8

**Published:** 2017-09-11

**Authors:** Lander Willem, Frederik Verelst, Joke Bilcke, Niel Hens, Philippe Beutels

**Affiliations:** 1 Centre for Health Economics Research & Modeling Infectious Diseases, Vaccine and Infectious Disease Institute, University of Antwerp, Antwerp, Belgium; 2 Interuniversity Institute for Biostatistics and statistical Bioinformatics, UHasselt, Hasselt Belgium; 3 School of Public Health and Community Medicine, The University of New South Wales, Sydney, Australia

**Keywords:** Individual-based, Agent-based, Mathematical epidemiology, Modeling, Emerging diseases, Endemic diseases, Transmission, Dynamics, Networks, ODD protocol

## Abstract

**Background:**

Individual-based models (IBMs) are useful to simulate events subject to stochasticity and/or heterogeneity, and have become well established to model the potential (re)emergence of pathogens (e.g., pandemic influenza, bioterrorism). Individual heterogeneity at the host and pathogen level is increasingly documented to influence transmission of endemic diseases and it is well understood that the final stages of elimination strategies for vaccine-preventable childhood diseases (e.g., polio, measles) are subject to stochasticity. Even so it appears IBMs for both these phenomena are not well established. We review a decade of IBM publications aiming to obtain insights in their advantages, pitfalls and rationale for use and to make recommendations facilitating knowledge transfer within and across disciplines.

**Methods:**

We systematically identified publications in Web of Science and PubMed from 2006-2015 based on title/abstract/keywords screening (and full-text if necessary) to retrieve topics, modeling purposes and general specifications. We extracted detailed modeling features from papers on established vaccine-preventable childhood diseases based on full-text screening.

**Results:**

We identified 698 papers, which applied an IBM for infectious disease transmission, and listed these in a reference database, describing their general characteristics. The diversity of disease-topics and overall publication frequency have increased over time (38 to 115 annual publications from 2006 to 2015). The inclusion of intervention strategies (8 to 52) and economic consequences (1 to 20) are increasing, to the detriment of purely theoretical explorations. Unfortunately, terminology used to describe IBMs is inconsistent and ambiguous. We retrieved 24 studies on a vaccine-preventable childhood disease (covering 7 different diseases), with publication frequency increasing from the first such study published in 2008. IBMs have been useful to explore heterogeneous between- and within-host interactions, but combined applications are still sparse. The amount of missing information on model characteristics and study design is remarkable.

**Conclusions:**

IBMs are suited to combine heterogeneous within- and between-host interactions, which offers many opportunities, especially to analyze targeted interventions for endemic infections. We advocate the exchange of (open-source) platforms and stress the need for consistent “branding”. Using (existing) conventions and reporting protocols would stimulate cross-fertilization between research groups and fields, and ultimately policy making in decades to come.

**Electronic supplementary material:**

The online version of this article (doi:10.1186/s12879-017-2699-8) contains supplementary material, which is available to authorized users.

## Background

Infectious diseases have substantial impact on public health, health care, macroeconomics and society. The availability of options to control and prevent the emergence, expansion or resurgence of pathogens warrants continuous evaluation using different methods. Mathematical models provide a powerful set of tools in this process, as timely, budgetary or ethically feasible alternatives are often lacking (e.g., school closure interventions or vaccine trials to study herd immunity effects) [1]. Even in countries or regions with high overall levels of vaccination coverage and herd immunity, sporadic outbreaks may still occur. For instance, in Europe, the flow of refugees through countries with ongoing large measles outbreaks (e.g., Bosnia-Herzegovina, Serbia) increased the risk of stochastic introduction events elsewhere [2]. Model-based evaluations can be useful to understand the behavioral mechanisms influencing the frequency, peak and duration of these outbreaks, with the aim to design (better) strategies to prevent them or minimize their impact [3].

Transmission dynamics of infectious diseases are usually modeled at the population-level with a compartmental model, and less frequently till now at the individual level with an individual-based model (IBM). A compartmental model tracks changes in compartments without specifying which individuals are involved [4]. Compartmentalization typically reflects health states relevant for transmission (e.g., susceptible, infectious and recovered), though more partitioning is possible according to age and/or other relevant host characteristics. Heterogeneous and temporal behavior is modeled through incorporation of relevant time-dependent social mixing, community structures and seasonality, relevant for infectious disease dynamics [5–7]. Process dynamics are captured in transition rates, representing the rate by which an average individual transitions between compartments. IBMs work bottom-up, with population-level behavior emerging from the interactions between autonomous individuals and their environment [8]. They allow the history of every individual to be tracked and network structures to be explicitly represented [4]. Each individual has a unique set of attributes or state variables that can change through time including spatial location, physiological traits and/or social behavior [9, 10]. As such, IBMs allow a high degree of heterogeneity for the creation, disappearance and movement of a finite collection of discrete interacting individuals [8, 11].

Deterministic models have been very useful to simulate the dynamics of endemic infections, but they are less suited to simulate events that are subject to chance [4]. For instance, the (non-)propagation of an infection in the initial stages of an emerging disease or in the final stages of elimination is dominated by individual heterogeneity and random events. The interplay between infectious disease dynamics and individual human behavior can be key to improve control efforts [12, 13]. Both compartmental and individual-based modeling approaches can simulate stochastic events. A compartmental model design, based on the epidemiological status of the population and known disease aspects, can be used in combination with stochastic and time-varying disease transmission rates [14]. As such, stochastic terms provide model flexibility to accommodate changes in the transmission rate that might occur due to unobserved processes. Remarkable progress has also been made with meta-population models to incorporate heterogeneous and temporal aspects by considering stochastic inter-population mobility [15]. For example, the Global Epidemic and Mobility (GLEaM) model, has been used to assess international travel restrictions during the 2009 influenza pandemic and the 2014-2016 Ebola outbreak [16–19]. Stochastic IBMs allow even more variation due to chance, which is especially of interest to study systems with small susceptible populations due to the context (e.g., a hospital or small island) or due to high population immunity (e.g., by routine childhood immunization programs).

Vaccination is one of the most effective tools to prevent infectious diseases and their consequences [20]. High immunization coverage is extremely important at the community level to protect patients who cannot be vaccinated due to medical reasons or age (e.g., the very young or very old). Indeed, also older age groups benefit from childhood immunization, for example the administration of conjugate pneumococcal vaccine to young children has had a substantial impact on adult pneumococcal disease [21]. However, low incidence of vaccine-preventable diseases in many high- and middle-income countries, often leads to the public perception of reduced severity and susceptibility [20]. Combined with rising concerns about real or perceived adverse events, the apparent absence of disease leads people to delay or refuse vaccinations more often [22]. Outbreaks of vaccine-preventable disease in countries with historically successful vaccination programs can take off in immigrant or unvaccinated pockets of susceptibles and potentially affect vulnerable groups such as infants and the immunocompromised [23]. Modeling the stochastic nature of transmission events in highly immunized populations with (clustered) heterogeneity in susceptibility can benefit from an IBM approach. To investigate the frequency and methods of such IBM applications, we focus on vaccine-preventable childhood diseases in a subsection of this review.

Different terminology has been used for individual-level models including agent-based model (ABM), cellular automata (CA), micro-simulation as well as more generic terms such as computer simulations and complex adaptive systems. A distinction in nomenclature can be designated by whether the simulation is based on nodes of a grid (as in a CA), or based on agents that are self-contained programs that collect information from their surroundings and have the autonomy and capacity to learn and adapt (ABM) [24]. These terms have been used interchangeably in the literature [25, 26]. Henceforth, we will use the overall term “IBM” to refer to the individual-level approach.

Describing the methodology of an IBM is more difficult compared to compartmental models, which often can be formulated in the general language of mathematics [27]. Published IBM methodology is often incomplete or ambiguous and therefore less accessible or reproducible [28]. In 2006, a board of 28 modelers developed and tested a generic format to document IBM research consisting of three blocks: Overview, Design concepts, and Details (ODD) [28]. The primary objective was to make model descriptions more understandable and complete. The “Overview” should provide readers the modeling focus, resolution and complexity based on the declaration of the model entities and the scheduling of the processes. The “Design Concept” describes the general approach to establish a link with emergence, the type of interactions and if/how stochasticity is considered. The “Details” section should contain all information required to completely reimplement the model and run the baseline simulations. In 2010, the ODD protocol has been revised and was used in at least 50 publications though still many papers lacked a standard approach to describe the IBM [29].

In this systematic review, we summarize and discuss IBM applications and terminology across different epidemiological disciplines, published between 2006 and 2015. We elaborate in general on the different modeling topics and purposes over time and identify research and data gaps. As indicated above we also focus on IBM research for childhood diseases with a long history of vaccination, i.e. on risk assessment and elimination strategies in heterogeneous settings with high population-immunity. We extract and discuss model characteristics such as the implementation of social mixing, demographic evolution over time, as well as the modeling platforms for IBMs. For these applications, we aimed to identify the rationale for an IBM and provide model characteristics and recommendations to enhance knowledge transfer across disciplines.

## Methods

Our search, extracting and reporting strategy is based on the evidence-based protocol PRISMA (Preferred Reporting Items for Systematic Reviews and Meta-Analyses) [30] and the Cochrane guidelines [31]. We use IBM as the overarching term for models at the individual-level, also noted as ABM, CA, micro-simulation, etc. We conducted a systematic review of studies using an IBM for infectious disease transmission, using this definitions based on the literature:

- **Infectious diseases:** “Caused by pathogenic microorganisms, such as bacteria, viruses, parasites or fungi; the diseases can be spread, directly or indirectly, from one person to another. Zoonotic diseases are infectious diseases of animals that can cause disease when transmitted to humans” [32].
- **Individual-based model:** “Computer simulation for the creation, disappearance and movement of a finite collection of interacting individuals or agents with unique attributes regarding spatial location, physiological traits and/or social behavior” [8–11, 25, 33].

### Search

We searched PubMed and Web of Science Core Collection using Endnote (X7.2.1) for English language articles published from January 2006 up to December 2015. Based on the listed definitions and exploratory searches, the following search query was used on January 3, 2016: “(model* OR simulat*) AND (agent-based OR individual-based OR individual-level OR multi-agent OR actor-based OR micro-simulation OR microsimulation OR cel* automata OR (stochastic AND individual*)) AND (disease OR infect* OR transmi* OR epidem*)”. Pubmed and Web of Science both ignore hyphens in the search query, so e.g., “individual based” and “agent based” were also retrieved. In line with Cochrane guidelines, eligibility criteria were agreed upon by four researchers (LW, JB, NH and PB, experienced in infectious disease and/or individual-based modeling) prior to screening. We included original research papers using an IBM with a focus on infectious disease transmission in humans. I.e., reviews and studies related to animal research, ecology, molecular biology and immunology were excluded. The screening on title/abstract/keywords and full-text if necessary was conducted by a single reviewer (LW), in consultation with co-authors in case of doubt.

### Model classification

For each study that met the eligibility criteria, LW and FV retrieved independently the topic (disease), the modeling purpose (methods, dynamics or interventions) and model specifications such as setting, economic analysis, reference data, open-source initiatives and sustainability based on model names. We classified the modeling purpose according to the following definitions: (1) methods: describing new approaches for IBM research by introducing modeling concepts, performance enhancements or emulation techniques; (2) dynamics: using a methodology to understand transmission dynamics and elaborate on the effect of model assumptions and parameter values on the results; (3) interventions: to evaluate intervention measures to inform policy makers, using a methodology and based on knowledge on the transmission dynamics. Studies for which LW and FV disagreed with respect to classification were discussed up to when agreement was reached.

### Full-text screening

To extract model characteristics and applications, a full-text screening was done in duplicate by LW and FV for papers on vaccine-preventable childhood diseases, defined as the diseases included in the immunization recommendations between birth and 15 months by the Centers for Disease Control and Prevention [34]. As such, we included diphtheria, *Haemophilus influenzae* type b, hepatitis A, hepatitis B, influenza, measles, meningococcus, mumps, pertussis, pneumococcus, polio, rotavirus, rubella, tetanus and varicella. Papers on influenza were excluded from the full-text analysis to focus on limited stochastic outbreaks in heterogeneous populations with high levels of herd immunity. For more info on forecasting influenza outbreaks, we refer to the systematic review by Nsoesie et al. [35]. For each full-text article, we listed the topic, the setting, model specifications (e.g., state variables, time horizon, step size), design of experiments (e.g., realizations, platform), the added value of an IBM compared to deterministic alternatives and the terminology.

## Results

Using the online databases PubMed and Web of Science, we identified 5520 unique articles published between 2006-2015 matching the search criteria listed in the “Methods” section. Our query included many general descriptions for IBMs and infectious disease transmission to decrease the number of false negative hits. Based on title, abstract and keyword screening with predefined eligibility criteria (see “Methods”), we excluded 4761 articles. More specifically, we excluded over 800 articles on a different topic (stock markets, oncology, engineering, non-human, etc.) and many more with a stochastic model but not at the individual-level. Other infectious disease IBMs did not include transmission events. We analyzed full-texts for 100 abstracts containing an unclear or incomplete model description and excluded 62 of them. Finally, we obtained 698 studies using an IBM to simulate infectious disease transmission. The adapted PRISMA diagram of the screening process with inclusion and exclusion criteria can be found in Additional file 1. In this main text, we describe and discuss general findings and provide the complete set of references with study characteristics as Additional file 2.

### Modeling purpose

**Fig. 1:**
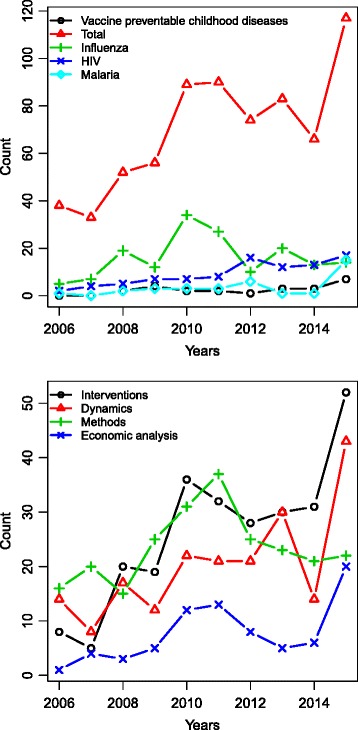
IBM studies published over time by topic (top) and purpose (bottom)

Among the 698 included studies, we observed an absolute increase in the annual number of IBM publications (38 to 115 from 2006 to 2016) and the diversity of disease-topics (Fig. 1). Most papers in our selection are on unspecified close-contact diseases (27%), closely followed by influenza (23%). Many studies in the latter group were published shortly after the 2009 H1N1 pandemic [36–38]. A similar event-related trend is observed for Ebola in 2015 [39, 40] and for bioterrorism subjects, with 13 studies between 2006 and 2013, expressing the rising concerns over smallpox [41], anthrax [42] and pneumonic plague [43]. Table 1 presents an overview of the different topics, modeling purposes and study characteristics. We observed that models for general close-contact diseases are mostly used to describe methodology and transmission dynamics. In contrast, many studies on influenza are conducted to control seasonal or pandemic outbreaks with vaccination programs or social distancing such as isolation and school closures [44, 45]. In recent years, we observe a shift for the use of IBMs from methodological (43% to 19%) to application and intervention-related purposes (21% to 44%). This is entangled in the rising number of articles on the transmission and control of human immunodeficiency virus (HIV), human papillomavirus (HPV), malaria, tuberculosis and methicillin-resistant *S. aureus*. Studies on sexually transmitted infections increasingly tend to evaluate screening strategies in the general population, compared to previous studies focusing on prevention measures for men who have sex with men or injecting drug users. We observed an accelerating trend in economic analyses using an IBM from 1 study in 2006 up to 20 in 2015. Malaria is the dominant topic for vector-borne disease models, covering drugs and vector control but also, more recently, potential malaria vaccination options [46–48]. Dengue has also been modeled using IBMs, though usually with the primary aim to understand the transmission dynamics, pathogenicity and epidemiology rather than to inform policy makers [49]. Many other diseases have also occasionally been modeled using IBMs including the respiratory syncytial virus [50] and cholera [51]. IBM studies on vaccine-preventable childhood diseases appeared in 2008 for measles and pneumococcus, accumulating to 24 studies by the end of 2015 covering meningococcus, varicella, polio, pertussis and hepatitis A (see “Full-text analysis” subsection for more details).

**Table 1:** Characteristics of IBMs studies for infectious disease transmission published from 2006 to 2015

Topic		Count	Purpose			Strategy				Economic
Pathogen	Type		Methods	Dynamics	Interventions	Vaccine	NPI	Drugs	Screening	Analysis
Unspecified Close-contact	General	186	95	77	14	5	9	1	-	4
Unspecified STI	General	9	3	5	1	1	1	-	1	-
Unspecified Vector-borne	General	7	7	-	-	-	-	-	-	-
Bioterrorism	General	6	3	1	2	-	2	1	-	1
Influenza	Viral	161	51	37	73	37	47	24	3	14
HIV	Viral	91	25	25	41	3	12	25	15	23
HPV	Viral	27	2	2	23	23	-	-	7	15
Hepatitis C	Viral	12	2	3	7	1	-	6	2	3
Ebola	Viral	8	-	5	3	1	3	2	-	-
SARS	Viral	8	4	3	1	1	-	-	1	-
Smallpox	Viral	7	-	-	7	7	3	-	3	-
Measles	Viral	5	1	1	3	3	-	-	1	-
Polio	Viral	4	1	2	1	1	-	-	-	-
HIV+Hepatitis C	Viral	3	1	1	1	-	1	-	-	-
HIV+HSV	Viral	3	-	1	2	1	-	1	-	1
Varicella Zoster	Viral	3	1	2	-	-	-	-	-	-
Respiratory syncytial virus	Viral	2	-	-	2	2	-	-	-	1
Acute hemorrhagic conjunctivitis	Viral	2	2	-	-	-	-	-	-	-
Hepatitis A	Viral	1	-	-	1	1	3	-	-	-
Norovirus	Viral	1	-	1	-	-	-	-	-	-
Malaria	Vector-borne	35	7	5	23	7	1-	11	3	5
Dengue	Vector-borne	13	4	5	4	2	3	-	-	-
Chikungunya	Vector-borne	1	-	-	1	-	1	-	-	-
Schistosoma	Parasitic	3	2	1	-	-	-	-	-	-
Wuchereria	Parasitic	3	2	-	1	-	1	1	-	-
Helminths	Parasitic	2	-	1	1	-	-	1	-	-
Onchocerca	Parasitic	2	-	-	2	-	-	2	-	1
Chagas disease	Parasitic	1	1	-	-	-	-	-	-	-
Toxocara	Parasitic	1	-	1	-	-	-	-	-	-
Cryptosporidium	Parasitic	1	1	-	-	-	-	-	-	-
Tuberculosis	Bacterial	26	8	6	12	-	-	9	9	4
MRSA	Bacterial	14	3	2	9	-	13	2	4	1
Chlamydia	Bacterial	7	3	-	4	1	-	-	3	1
Nosocomial infections	Bacterial	7	3	2	2	1	4	-	-	-
Syphilis	Bacterial	6	-	-	6	-	2	1	4	1
Pneumococcus	Bacterial	5	1	1	3	2	1	-	-	-
Cholera	Bacterial	4	-	4	-	-	-	-	-	-
Lepra	Bacterial	4	-	2	2	1	-	2	1	-
Gonorrhoea	Bacterial	3	-	1	2	1	-	-	1	-
Clostridium difficile	bacterial	3	-	-	3	-	7	2	2	-
Pertussis	bacterial	3	-	1	2	2	-	-	-	1
Meningococcus	bacterial	3	1	1	1	1	-	-	-	-
Acinetobacter baumannii	bacterial	1	-	-	1	-	2	-	-	-
Enterococcus	bacterial	1	-	1	-	-	-	-	-	-
Typhoid	bacterial	1	-	1	-	-	-	-	-	-
Mycobacterium ulcerans	bacterial	1	-	1	-	-	-	-	-	1
Foot and mouth disease	zoonose	1	1	-	-	-	-	-	-	-
Total		698	235	202	261	105	125	91	60	77

Each study was assigned one purpose, which is cumulative from methods, dynamics to intervention (e.g., studies about interventions can also describe dynamics and methods). The category “NPI” includes all non-pharmaceutical intervention strategies such as social distancing, school closure and improving standards of living and (hand-)hygiene. HIV: human immunodeficiency virus, HPV: human papillomavirus, HSV: herpes simplex virus, MRSA: methicillin-resistant *S. aureus*, STI: sexually transmitted infection

Methodological papers, not applied to a specified close-contact infection, mostly describe the conceptual usage of an IBM to simulate heterogeneous disease dynamics and targeted intervention strategies. Other studies were published on validation procedures [52, 53], performance issues [54, 55] and emulation to improve rapid policy making in various settings [55–58]. Models and model output have been calibrated and validated with observed incidence and (sero)prevalence data [49, 59, 60] but also with data generated by other models, such as deterministic ordinary differential equation models [61] or meta-populations models [62].

As supplementary analysis, we explored the relative number of studies over time that have or have not used an IBM to model infectious disease transmission (described in Additional file 3). We performed additional literature queries considering the number of records in Web of Science as a proxy for the effective number of modeling studies and a constant fraction of false positives and negatives over time. As such, we observed that the yearly number of published IBM related studies tends to increase more rapidly since 2006 compared to the annual publications on modeling infectious disease transmission in general.

### Terminology

We observed a variety of descriptions for models simulating transmission events between humans at the individual-level. Table 2 illustrates the presence of query terms in all unique hits and for the selected subset of IBMs for infectious disease transmission. The positive predictive value represents the proportion of positive results that are truly positive (i.e. the proportion of query records included after screening). We also estimated the sensitivity, namely the probability of detection, as the proportion of positives that are correctly identified as such. Of the 698 included studies, 12 did not contain “model” in their title, abstract or keywords. To describe the individual-level characteristics, ABM and IBM were mainly used, followed by CA or micro-simulation (though with different spelling variations). Other terminology that covered our definitions included “individual-level model” [63], “individually based SIR model” [64], “small world network” [65], “large-scale stochastic simulation” [66], “equation free approach” [67] or other variants of “stochastic models” [36, 68]. General keywords gave many false positive hits though still resulted in 124 papers that did not use the most common terminology in their abstract, title or keywords. None of our disease related query terms were used by the complete set of IBM papers on infectious disease transmission and a low positive predicted value was observed. Firstly, the term “disease” is also valid for chronic and lifestyle diseases. Secondly, we needed to include general terms such as “transmi*” or “epidem*” to capture papers only describing their specific disease topic like influenza [69] or dengue [70]. Unfortunately, “transmi*” caused many false positive hits for research on power markets, sensors and information networks.

**Table 2:** Terminology in abstract, title and keywords from all unique query hits and in the included IBM modeling studies for infectious disease transmission. One article can contain several terms

	All hits	Included articles	Positive predicted value	Sensitivity
Model*	5271	686	0.130	0.983
Simulat*	2318	504	0.217	0.722
Agent-based	969	251	0.259	0.360
Individual-based	616	241	0.391	0.345
Micro-simulation	396	54	0.136	0.077
Cel* automata	249	62	0.249	0.089
Other individual-level related terms (see Methods)	3367	124	0.037	0.178
Disease	2791	445	0.159	0.638
Infect*	1939	553	0.285	0.792
Transmi*	1847	441	0.239	0.632
Epidem*	3029	521	0.172	0.746
Disease OR infect*	3564	629	0.176	0.901
Infect* AND disease AND transmi*	628	252	0.401	0.361
TOTAL	5520	698	0.126	1.000

The asterisk (*) is used in the search as a wildcard and represents any group of characters, including no character

### Modeling group diversity and branding

Based on their acronym, some models were identified as having been applied multiple times, for example STDSIM [71], EPISIMS [72], EMOD [73], ONCHOSIM [74], HPV-ADVISE [75], FRED [76] and the Openmalaria platform [77]. This non-exhaustive list covers models for airborne, sexually transmitted, parasitic and vector-borne diseases. With such consistent acronyms, one can link studies for different diseases, such as STDSIM developed for HIV [71] but used for HPV [78] and herpes simplex [79] or FRED implemented for influenza [76] and recently used for measles [80], or EMOD used for HIV [81] and malaria [82]. In addition, we identified studies that were published by the same authors but links with previous research were not mentioned, at least not in the abstract. Based on authorship, research institute and project names, we could also link other studies to the Openmalaria platform [77] and FRED [76]. Providing IBM code open-source to the research community is not common practice but exists, for example with FluTE [36] and FRED [76].

### Full-text analysis

We analyzed 24 full-text articles on vaccine-preventable childhood diseases, excluding influenza to focus on limited stochastic outbreaks in heterogeneous populations and given the recent systematic review for influenza by Nsoesie et al. [35]. The articles covered transmission dynamics for hepatitis A, measles, meningococcus, pertussis, pneumococcus, polio and varicella. Our search did not yield studies for diptheria, *Haemophilus influenzae* type b, hepatitis B, mumps, rotavirus, rubella or tetanus. In the remainder of this section, we summarize the main findings from the full-text analysis, the per-study details of which can also be found in Table 3.

**Table 3:** Design of IBM studies on vaccine-preventable childhood diseases, excluding influenza

Reference	Topic, setting	Purpose	State variables	Population	Time horizon, step size	Realiza-tions	Platform	Reason IBM	Terminology
Grais et al. [91]	Measles, Niger	Reactive vaccination	Age, social mixing patterns, location	346.254 people	1 year, per day	1.000x	-	Spatio-temporal interventions and coverage	IBM
Perez and Dragicevic [24]	Measles, Canada	Dynamics in spatial context	Social mixing patterns	1.000 people	60 days, per hour	1x	RepastS	Spatio-temporal analysis	ABM
Liu et al. [80]	Measles, USA	Reactive vaccination and contact tracing	Age, social mixing patterns, compliance	118.261 people	1 year, per day	256x	C++ (FRED)	Contact tracing and clustering	ABM
Marguta and Parisi [84]	Measles, UK	Dynamics using detailed mobility patterns	Preferred locations	“British Isles”	60 years, per day	100x	-	Mobility patterns	IBM
Thompson and Kisjes [92]	Measles, USA Amish	Outbreak response in connected under-vaccinated subpopulations	Age, gender, social mixing patterns, conservatism, compliance	280.000 people (dynamic)	1 year, per 30 min	1000x	Netlogo	Clustering	IBM
Martinez et al. [85]	Meningococcus, Grid	Dynamics with immunity	Network location	1.000 people (static)	60 days, per day	1x	Mathe-matica	Spatio-temporal analysis	CA
Pérez-Breva et al. [99]	Meningococcus, Spain	Vaccination	Age, serotype	1 million people (dynamic)	36 years, per month	-	-	Serotype dynamics with spatial analysis	ABM
Poore and Bauch [101]	Meningococcus, Canada	Vaccination and serotype groups	Age, serotype	- (dynamic)	300 years, per month	50x	-	Serotype dynamics with spatial analysis	ABM
Monteiro et al. [86]	Varicella, USA	Dynamics and parameter fitting	Network position, neighbors	1 million people	11 years, per month	-	-	Spatio-temporal analysis	CA
Silhol and Boëlle [88]	Varicella, Corsica	Dynamics and parameter fitting	Age, social mixing patterns	35.000 children (dynamic)	100 years, per day	500x	-	Spatio-temporal analysis with clustering	ABM, IBM
Ogunjimi et al. [87]	Varicella, Belgium	Dynamics and parameter fitting	Age, cellular mediated immunity	998.400 people (dynamic)	320 years, per week	3x	MATLAB	Within-host cellular immunity	IBM
Ajelli and Merler [103]	Hepatitis A, Italy	Household dynamics, vaccination, NPI	Age, social mixing patterns	5.701.931 people (dynamic)	50 years, per week	-	-	Clustering and assessment of real-world interventions	IBM
Karlsson et al. [102]	Pneumococcus, Sweden	NPI	Age, social mixing patterns	25.000 people	15 years, per week	100x	MATLAB	Spatio-temporal analysis	IBM
Saito et al. [83]	Pneumococcus, Grid	Dynamics with antibiotics	Network location, social mixing behavior	2.500 people	400 days, per day	100x	-	Spatio-temporal analysis	CA
Choi et al. [98]	Pneumococcus, UK	Vaccination	Serotype	48 million people (dynamic)	20 years, per week	10x	MATLAB	Serotype dynamics with spatial analysis	IBM
Flasche et al. [97]	Pneumococcus, UK	Vaccination	Age, serotype	243.792 people (dynamic)	30 years, per day	-	C++ (on request)	Serotype dynamics with spatial analysis	IBM
Nurhonen et al. [100]	Pneumococcus, Finland	Vaccination and serotype replacement	Age, social mixing patterns, serotype	100.000 people (dynamic)	100 years, per day	50x	C++	Serotype dynamics with spatial analysis	ABM, IBM, micro-simulation
Rahmandad et al. [89]	Polio, Low-income country	Dynamics	Age, social mixing patterns	100.000 people (dynamic)	2000 days, per day	1000x	AnyLogic	Spatio-temporal analysis	ABM, IBM
Kisjes et al. [23]	Polio, USA Amish	Dynamics in connected under-vaccinated subpopulations	Age, gender, social mixing patterns	276.000 people (dynamic)	3 years, per 30 min	1000x	Netlogo	Clustering	IBM
Wagner et al. [93]	Polio, Nigeria	Expanded age group vaccination programs	Age, gender, risk factors	300.000 people (dynamic)	40 years, event-driven	-	C++ (EMOD)	Spatio-temporal risk factors	IBM
Kim and Rho [96]	Polio (vaccine-derived), Grid	Dynamics with immunity and vaccine-related side effects	Network location	100.000 people	25 years, per day	100x	-	Spatio-temporal analysis	IBM
Greer and Fisman [94]	Pertussis, USA	Booster vaccination programs in hospital setting	Social mixing behavior	38 patients with health care workers and family	3 month, per day	1000x	AnyLogic	Spatio-temporal analysis	ABM
de Vries et al. [95]	Pertussis, The Netherlands	Universal booster vaccination programs	Age	150.000 people (dynamic)	25 years, event-driven	20x	Arena	Individual stochastic disease burden	IBM
Sanstead et al. [90]	Pertussis, USA	Dynamics	Age	400.000 people	-, per day	100x	NetLogo	Within-host dynamics with spatial analysis	ABM

Studies are listed by topic. The state variables express the individual heterogeneity next to health-states (e.g., SIR, SIRV,... etc.). If multiple experiments are described, the maximal time horizon, minimal step size and maximum number of realizations are presented. A “dynamic population” considers next to health state also socio-demographical changes over time, such as aging and household alterations. NPI: all non-pharmaceutical intervention strategies, “-”: unknown

#### Purpose

We retrieved 2 papers that explored methodology to incorporate heterogeneous interactions in a (geo)spatial context [24, 83]. Eight papers elaborated on transmission dynamics, focusing on the influence of social mixing patterns or within-host dynamics [23, 84–90]. Additionally, 14 studies modeled intervention strategies to mitigate infectious disease outbreaks. The majority (12/14) of these studies modeled vaccination campaigns targeting general [80, 91] or insufficiently immunized subgroups [92, 93], expanding booster campaigns [94, 95], the occurrence of rare adverse events such as vaccine-induced polio [96] and serotype carriage and replacement [97–101]. Two other studies on intervention strategies evaluated social distancing options and adaptive social contact behavior [102, 103].

#### Setting

We found papers modeling a theoretical grid [83, 85, 96] or a generic “low income setting” [89]. The study population of the other papers did not exceed a single country, and ranged from a North American [23, 24, 80, 86, 90, 92, 94, 101] to a European [84, 87, 88, 95, 97–100, 102, 103] or African [91, 93] country.

#### State variables

The lowest-level entity in each model was a “person” and the minimum characteristic was the health state. Depending on the research questions, also heterogeneity for age, gender, spatial location, social mixing behavior [103], compliance to reactive strategies [92], serotype carriage [99] and cellular mediated immunity [87] were incorporated. Social mixing behavior and transmission events were modeled in one unified population [87, 97] and/or within specific social contact clusters such as households, schools, workplaces and communities [84, 103], sometimes in combination with occasional long distance trips [92].

#### Population

The population sizes ranged from 38 infants in a hospital setting [94] up to 48 million inhabitants of England and Wales [98]. The dynamics regarding age and social mixing in the population were modeled static (i.e. constant) [85] or dynamic (with ageing, mortality, newborns, weddings) [92, 100]. Ajelli and Merler [103] were exceptional in that they provided an explicit approach to model household dynamics over time to enable IBM simulations on long time-scales.

#### Time horizon

We observed a spectrum of time horizons from 60 days [24] up to 320 years [87]. The step size was mostly one day (e.g., [83, 88, 91]) but ranged from 30 minutes [23] up to one month [86]. Two event-driven models had no fixed time steps [93, 95].

#### Realizations

For stochastic IBMs, one initial condition can lead to different outcomes so multiple realizations are highly recommended. The number of realizations for each parameter set to quantify the uncertainty on the results varied in our search from 3x [87], 10x [98] and 100x [90] up to 1000x [89]. For five papers, we were not able to retrieve the number of realizations [86, 93, 97, 99, 103].

#### Platform

We distinguished a category of papers using mathematical software such as MATLAB^®;^ [102] and Mathematica^®;^ [85]. Others used more explicit modeling platforms for IBMs such as NetLogo [23, 90], RepastS [24] and AnyLogic^®;^ [94]. One model was implemented in Arena, which is specific software for discrete-event simulations [104]. Four studies reported a model implemented in C++ [80, 93, 97, 100].

#### Reason IBM

We discerned 3 main reasons for choosing an IBM for these childhood diseases. Firstly, to model heterogeneous between-host interactions regarding social mixing behavior, age, demography, clustering, compliance to mitigation strategies and spatial distribution (e.g., [85, 88, 91]). Secondly, to model heterogeneous within-host processes in combination with between-host interactions (e.g., [87, 98, 99]). For instance, Choi et al. [98] analyzed serotype replacement and developed an IBM to track the multitude of possible vaccine states and dose combinations, which was too complex to capture in a compartmental model framework. Thirdly, to obtain stochastic individual-level information on the disease burden to inform economic analysis or other post-processing [95].

#### Terminology

Of the 24 articles, 12 used only “IBM” to denote their individual-level transmission model. Six papers used “ABM” and 3 “CA”. Nurhonen et al. [100] used the terms “IBM”, “ABM” and micro-simulation interchangeably. Silhol and Boelle [88] and Rahmandad et al. [89] used “IBM” and “ABM”.

#### Model performance

Only Rahmandad et al. [89] defined model requirements and performance. They reported runtimes and stated that specialized computer clusters were required to simulate very large populations. To set up the scale-free network, 30 minutes were required on an Intel Core^®;^ 2 CPU 6400@2.13 GHz desktop. The runtimes to model transmission dynamics scaled with population size. A few papers mentioned that their results were obtained on a cluster [87, 89, 100], without providing details.

#### Other

The amount of missing information on the platform or other technical details is noteworthy, especially when the model is not described elsewhere or open-source. Two papers provide a model name, FRED [80] and EMOD [93], and one states that the source code is available on request [97]. In some papers [83, 95, 100], model characteristics such as population size, time horizon, step size or number of realizations had to be retrieved from the “Results” or “Discussion” sections or from figure captions.

## Discussion

The number of published IBMs for infectious disease transmission and the diversity of disease topics are increasing. Our systematic search identified 698 unique papers between 2006 and 2015. Most included articles were applied to unspecified close-contact infections or to influenza, though IBMs for other air-, saliva-, vector-borne and sexually transmitted infections are emerging. Methods for vector-borne diseases have been described for malaria and dengue and could guide future research. Especially, IBM applications on chikungunya and zika are expected over the next decade given the growing geographical expansion of their common vectors [49, 105]. Also screening and (non-)pharmaceutical intervention strategies have not been fully explored with IBMs for many diseases. Given the heterogeneous nature of bio-medical and socioeconomic data and the accelerating health care expenditures, IBMs become progressively useful to inform policy makers, particularly in combination with efficiency and equity analyses [106, 107]. There are relatively few papers with an IBM for stochastic outbreak analysis under high vaccination coverage, for example for vaccine-preventable childhood diseases. For measles, it was shown that stochastic fluctuations around the endemic equilibrium in populations with high vaccination coverage could cause recurrent epidemics [84]. We expect future research to focus more on these topics with IBMs in combination with increasing global mobility, urbanization, climate change, disease elimination efforts and vaccine skepticism [2]. Customization of health care is one way to mitigate these stochastic epidemics with medical interventions tailored to the individual patient. The rising transition towards precision medicine needs to be informed with studies on the individual-level to capture spatio-temporal heterogeneity.

Modeling frameworks, such as STDSIM [71], EMOD [73] and EPISIMS [72] exist but are limited in that their application depends heavily on specific input data. Indeed, it is difficult to create or maintain generic models that incorporate many modeling options and still manage the computational burden. Nonetheless, given the high programming burden, transparent reuse of models increases confidence in their approach and generated results. Making IBM code open-source (e.g., FluTE [36] and FRED [76]) is also useful to validate model outcomes, to inspire future modeling projects [55] and to expand model exploration [108]. Consistent “branding” of the IBM, with a proper acronym, is practical to link studies and consolidate intellectual ownership of freely accessible source code.

Regarding the simulation platform, mathematical software (e.g., MATLAB^®;^) enables many embedded features and is user-friendly but currently lacks specific modules for IBMs. Integrated platforms such as RepastS [109] and Netlogo [25, 110], are used by others and can be practical and straightforward but cannot fulfill all requirements of the inherent heterogeneity and computational burden of IBMs. A third option is the low-level programming language C++, which enables high-performance code but requires high-level programming skills to efficiently manage the model logic and memory usage. Given the computational and implementation burden [84, 89], close interaction with computer sciences is required. Nonetheless, good-practice programming with version control, regression testing and benchmarking is rarely described [108, 111].

Although runtimes are inherent to model implementation and computer hardware, presenting the order of magnitude of runtimes and memory requirements could be useful for other researchers. Details on model performance and computational burden were usually lacking in our selection of full-text papers. In our total set of IBM papers, we found 2 examples on the computational burden of their IBM in C++ [36, 76]. An influenza simulation with FLUTE [36] uses approximately 80 megabytes of memory per million simulated individuals. Simulating an epidemic in a population of 10 million people can take up to two hours (on a single processor on an Intel^®;^ Core Duo T9400), but it may take only seconds if the virus is not highly transmissible or if there are effective interventions [36]. With 750 - 1000 megabytes of memory required per million simulated individuals, FRED’s computational burden [76] is about ten times larger. Simulations for the H1N1 pandemic in a population of 1 million people takes less than two minutes on a typical dual-core laptop computer (in 2013) but the runtime will vary depending on the number of individuals infected during the epidemic and depending on which optional features are activated. Unfortunately, computational performance is a significant aspect of a simulator’s usefulness. Investment in performance optimization is required to achieve the full potential of current high-performance workstations [108]. This seems most feasible using open-source software, as it allows more researchers to contribute to optimization and to leverage on the existing - and ever expanding - IBM knowledge base, thus enabling a cyclic process of innovation and optimization.

Time horizons and modeling step sizes in the full-text articles were diverse and are subject to disease characteristics and research objectives. There is no standard approach on the number of stochastic realizations, which seems model specific and requires sensitivity analysis. Models focusing on key factors of between-host dynamics in large populations with homogeneous mixing [87] will not produce much stochastic variability and require fewer realizations compared to simulations combining complex social mixing clusters, adaptive behavior, within-host dynamics and medical backgrounds [80, 91, 92]. One of the most frequent criticisms of IBMs is that “they can be calibrated to say anything” [25]. This is partly a result of not capturing the difference between the calibration of IBMs and equation-based models. The latter have usually fewer parameters, which have to be evaluated by calibrating the full model to observed data [25]. IBMs, in contrast, are constructed bottom-up, which allows to select parameters independently based on census data, mobility patterns, serotype distribution, social contact behavior, natural history of a disease, etc. As such, a limited number of particularly uncertain parameters has to be calibrated by fitting the model to observed prevalence and/or incidence of disease states [25]. IBM calibration has been performed with genetic algorithms [86], maximum likelihood [88] or Bayesian procedures with Markov Chain Monte Carlo sampling [53]. A limitation of the IBM approach is that the basic reproduction number (R_0_), corresponding to the number of secondary cases caused by a single (typical) infection in a totally susceptible population, cannot be attributed directly but has to be derived from model output. R_0_ has been estimated in IBM studies [23, 36, 76, 88, 108, 112] by the average number of secondary cases from a randomly selected individual in a fully susceptible model population based on multiple realizations. Parameterization and calibration needs to be documented well. Model presentation should preferably be accompanied by an assessment of the goodness of fit to observed data [4]. Another convincing way to show that your ABM has been calibrated without bias and produces useful general results is to analyze it thoroughly after calibration [25]. Ideally, each model should be analyzed systematically to understand the impact of model assumptions and parameters on the results [55]. Parameter values can be drawn from a pre-computed design (e.g., Latin Hypercube) or at random from a distribution. Emulation techniques are promising to capture complex simulators’ behavior in order to improve engaged and perhaps more rapid policy making [55–58]. Given the lack of standards, it is crucial to fully describe the methods and experimental design in the context of the model [4]. Unfortunately, we were not able to recapture all model characteristics and study designs from our full-text subset. This stresses the need for the ODD protocol with shorthand conventions and a syntax that modelers can understand intuitively such that the methodology can be converted directly into an executable simulator [28].

The terminology to describe individual-level models and infectious diseases was inconsistent and curtail efficient knowledge transfer. For example, a systematic review in 2015 on IBMs for non-communicable diseases [33] searched only with the terms “agent-based” and “individual-based” to dramatically reduce the number of false positive hits. To assist future research, it is crucial to use the same semantics for IBM studies across disciplines. The introduction of the Medical Subject Headings (MeSH) controlled vocabulary [113] is a huge step forward but is limited to PubMed and does not (yet) contain fixed terms for simulation models at the individual-level. With this review, we seek to provide keywords to the IBM community and a definition for individual-based modeling as *“computer simulation for the creation, disappearance and movement of a finite collection of interacting individuals or agents with unique attributes regarding spatial location, physiological traits and/or social behavior”* [8–11, 25, 33]. The overall term IBM refers to the individual-level approach based on a fundamental philosophy of methodological individualism, which advocates a focus on the uniqueness of individuals and their interactions. Further subcategories can be used according to whether locations are static (as in CAs) or individuals act autonomously (as in ABMs). The standard incorporation of the overarching term “individual-based model” in the abstract or keywords would greatly improve current and future systematic searches in large electronic databases.

One could argue that our recommendations are constrained since they are based on title, abstract and keyword screening. For example, a frequently cited article on pandemic influenza by Ferguson et al. [114] was not retrieved by our search since it has none of the IBM terms in its title, abstract or keywords. The model is described as a *“large-scale epidemic simulation”* in the abstract although the first sentence of the introduction reads *“We parameterize an individual-based model of pandemic influenza transmission...”*. This example could be seen as a confirmation of the inconsistencies and limitations of current article archiving practice. A similar remark can be made for our disease related search terms but we believe we used the most relevant keywords and can only recommend future research to include also general disease-related terminology. If our selection was identifiable by searching on “infectious AND disease AND transmission”, this would be a substantial improvement, in contrast to the current 40% of our selection. The restriction to only include papers published between 2006 and 2015 might be considered a minor limitation by the time the current paper is published. The final fully included year was chosen mainly for practical reasons, at the time of completing this labour-intensive review in early 2016. We are convinced that a review over a decade (an intuitively appealing period for review) is highly informative to understand the evolution of this field and to adequately guide future research. We had no intention to present a complete review of all the IBM papers we systematically identified, but we provide all included references in a searchable database enabling others to conduct more specific literature reviews. Clearly, our database can be updated using the discussed insights on search methodology and keywords.

## Conclusion

We systematically reviewed a decade of recent literature on infectious disease transmission IBMs and propose a common terminology to facilitate knowledge transfer within and across disciplines. IBMs have already been useful to explore heterogeneous between-host interactions both with and without unique within-host (dynamic) processes. The number of IBMs to study transmission and control of HIV, HPV, malaria and tuberculosis is increasing. The combination of targeted screening and vaccination strategies with economic evaluations is promising for the near future. Emerging diseases are the dominant applications in infectious disease IBMs. Notwithstanding, similar models are required for endemic diseases, such as vaccine-preventable childhood diseases, to capture stochastic and heterogeneous characteristics, which are especially relevant in the final stages of elimination. We provide 698 unique references published between 2006-2015 with study characteristics to inform the research community across topics and terminology. We recommend cooperation in open-source projects and adhering to the ODD protocol, which enables modelers to describe their IBM using a common syntax. Common model-names enhance the research community’s ability to grasp common features between models, and discover opportunities for further model improvements. Transfer of expertise on IBMs is required to capitalize future research opportunities, which is facilitated through the increasing availability of individual-level data and the rising interest for precision medicine. In this respect, the combination of screening and targeted vaccination strategies with economic evaluations seems an interesting future prospect.

## Additional files

Additional file 1 PRISMA flow diagram. Adapted PRISMA flow diagram of the systematic review process. (PDF 52 kb)

Additional file 2 Reference list. List of the included studies using an individual-based model to study infectious disease transmission. References are sorted by topic and disease and listed together with their terminology, modeling purpose, (non)inclusion of an economic analysis, intervention strategy and methodology classification. (CSV 183 kb)

Additional file 3 The relative number of IBM studies over time. Supporting information on additional literature searches to estimate the relative number of IBM studies for infectious disease transmission over time compared to modeling studies for infectious disease transmission in general. (PDF 93 kb)

## Abbreviations

ABM: Agent-based model
CA: Cellular automata
HIV: Human immunodeficiency virus
HPV: Human papillomavirus
IBM: Individual-based model
ODD: Overview, Design concepts, and Details (protocol)
