# Supplementary figures and images for: Lessons from a decade of individual-based models for infectious disease transmission: a systematic review (2006-2015)
Source: BMC Infect Dis. 2017 Sep 11;17:612. doi: 10.1186/s12879-017-2699-8 (PMC5594572; doi:10.1186/s12879-017-2699-8)

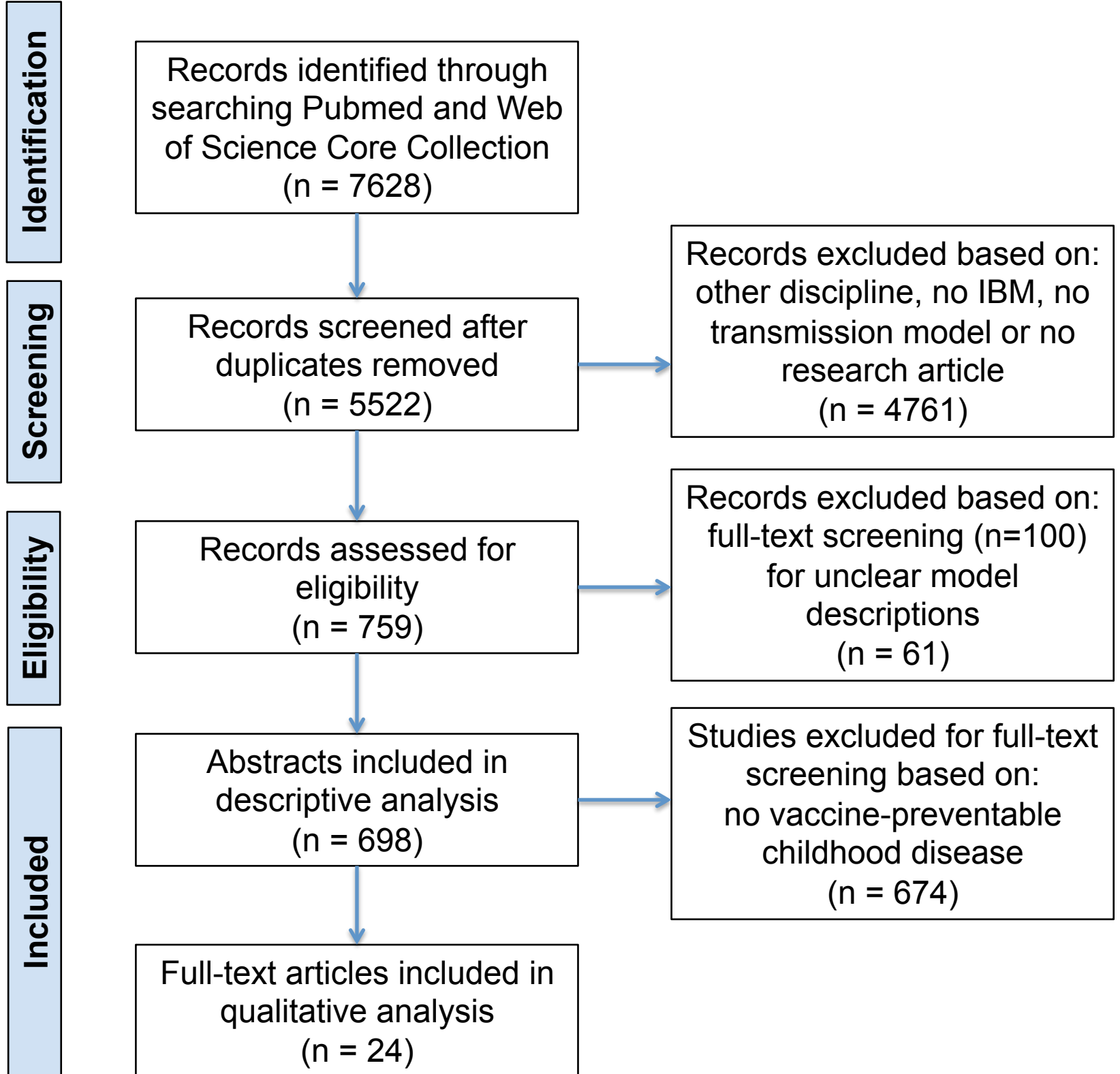

Supplement: Supplementary file 1 — PRISMA flow diagram. Adapted PRISMA flow diagram of the systematic review process. (PDF 52 kb) [file 12879_2017_2699_MOESM1_ESM.pdf]
