# Supplementary material for: Lessons from a decade of individual-based models for infectious disease transmission: a systematic review (2006-2015)
Source: BMC Infect Dis. 2017 Sep 11;17:612. doi: 10.1186/s12879-017-2699-8 (PMC5594572; doi:10.1186/s12879-017-2699-8)
Supplement: Supplementary file 3 — The relative number of IBM studies over time. Supporting information on additional literature searches to estimate the relative number of IBM studies for infectious disease transmission over time compared to modeling studies for infectious disease transmission in general. (PDF 93 kb) [file 12879_2017_2699_MOESM3_ESM.pdf]

Lessons from a decade of individual-based models for infectious disease transmission: a systematic review (2006-2015)

Supplementary information

Lander Willem[1*], Frederik Verelst[1], Joke Bilcke[1], Niel Hens[1,2] and Philippe Beutels[1,3]

[1] Centre for Health Economics Research & Modeling Infectious Diseases,Vaccine and Infectious Disease Institute, University of Antwerp, Antwerp, Belgium. [2] Interuniversity Institute for Biostatistics and statistical Bioinformatics, UHasselt, Hasselt, Belgium. [3] School of Public Health and Community Medicine, The University of New South Wales, Sydney, Australia. [*]Correspondence: lander.willem@uantwerp.be

Introduction

In the main text, we reported an increase in the yearly number of published individual-based models (IBMs) for infectious disease transmission over time. In this appendix, we compare this trend with the annual number of published transmission models for infectious diseases in general.

We consider the number of records in Web of Science as a proxy for the effective number of modeling studies, under the assumption of a constant fraction of false positives and negatives over time. As such, we can compare results from a query for "transmission models for infectious diseases" with the results from a query for "IBMs for infectious disease transmission". The latter is similar to the query we used in the main text.

Methods

We searched Web of Science Core Collection queries on June 21, 2017, for English language articles published from January 1990 up to December 2015 via the online tool https://apps.webofknowledge.com.

- **QUERY 1: [model] & [disease]**

(model OR simulat*)*
AND
(disease OR infect OR transmi* OR epidem*)*

- **QUERY 2: [model] & [disease] & [IBM]**

(model OR simulat*)*
AND
(disease OR infect OR transmi* OR epidem*)*
AND
(agent-based OR individual-based OR individual-levelOR multi-agent OR actor-based OR micro-simulation OR microsimulation OR cel automata OR (stochastic AND individual*))*

Results

In total, we obtained 4.657.512 results with the “[model] & [disease]” query and 5.690 records with the “[model] & [disease] & [IBM]” query. Figure S1 presents the annual number of records in Web of Science over time between 1990 and 2015. We observed two increasing trends. Since 2006, the yearly number of IBM related studies tends to increase more rapidly compared to the annual publications on infectious disease transmission modeling in general. As such, we conclude that the relative number of transmission modeling studies using an IBM and indexed in Web of Science Core Collection has increased in the last decade.

Discussion

More info on the positive predicted value and sensitivity of both queries is missing and falls outside the scope of this review. Hence, we cannot calculate the absolute fraction of IBM studies compared to the total number of transmission modeling studies. The fraction of 0.2 % in 2015 (759 “IBM studies” / 371.245 “modeling studies”) has no meaning if both queries have a different positive predicted value. We can only compare general trends, as long as we assume that the fraction of false positives and negatives is constant over time.

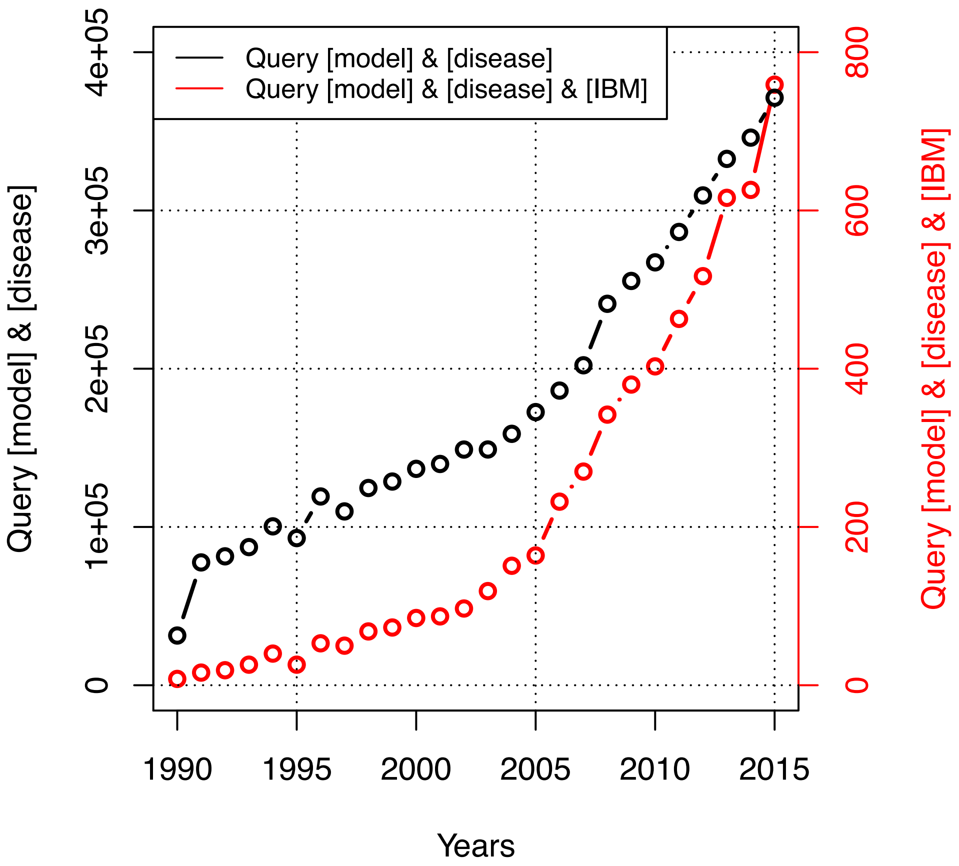

Figure S1. Number of records in Web of Science Core Collection over time derived with a query for “infectious disease transmission models” (left axis) and a query for “individual-based infectious disease transmission models” (right axis).
